# Expression of estrogen and progesterone receptors in astrocytomas: a literature review

**DOI:** 10.6061/clinics/2016(08)12

**Published:** 2016-08

**Authors:** Cléciton Braga Tavares, Francisca das Chagas Sheyla Almeida Gomes-Braga, Danylo Rafhael Costa-Silva, Carla Solange Escórcio-Dourado, Umbelina Soares Borges, Airton Mendes Conde, Maria da Conceição Barros-Oliveira, Emerson Brandão Sousa, Lorena da Rocha Barros, Luana Mota Martins, Gil Facina, Benedito Borges da-Silva

**Affiliations:** I Universidade Federal do Piauí, Programa de Pós-graduação de Ciência e Saúde, Teresina/PI, Brazil; II Hospital São Marcos, Teresina/PI, Brazil; III Universidade Federal do Piauí, Departmento de Mastologia, Teresina/PI, Brazil

**Keywords:** Estrogen Receptor Alpha, Estrogen Receptor Beta, Progesterone Receptor, Astrocytoma

## Abstract

Gliomas are the most common type of primary central nervous system neoplasm. Astrocytomas are the most prevalent type of glioma and these tumors may be influenced by sex steroid hormones. A literature review for the presence of estrogen and progesterone receptors in astrocytomas was conducted in the PubMed database using the following MeSH terms: “*estrogen receptor beta” OR* “*estrogen receptor alpha” OR “estrogen receptor antagonists” OR “progesterone receptors” OR “astrocytoma” OR “glioma” OR* “*glioblastoma”*. Among the 111 articles identified, 13 studies met our inclusion criteria. The majority of reports showed the presence of estrogen and progesterone receptors in astrocytomas. Overall, higher tumor grades were associated with decreased estrogen receptor expression and increased progesterone receptor expression.

## INTRODUCTION

Gliomas are the most common type of primary central nervous system tumor and astrocytomas are the most prevalent type of glioma. According to the World Health Organization (WHO), these tumors may be classified into two types: low-grade or benign (grades 1 and 2) and high-grade or malignant (grades 3 and 4) 1,2.

High-grade glial tumors are the most common primary malignant tumors of the central nervous system in adults. Despite appropriate treatment with surgical excision, chemotherapy and radiation therapy, the prognosis is poor 3,4. Nevertheless, improved survival seems to depend on the understanding and manipulation of pathways that regulate aberrant tumor growth 1.

Estrogens are steroid hormones that exert important effects on the reproductive and gastrointestinal systems, mammary glands, skeletal and immune systems, and even the central nervous system. The majority of estrogen effects are mediated mainly by estrogen alpha (ERα) and beta (ERβ) receptors 5,6.

Progesterone participates in the regulation of several reproductive processes, including ovulation and sexual behavior. In synergism with estrogen, progesterone also influences neuronal excitability, learning and neoplastic proliferation of glial cells. These progesterone effects result mainly from the interaction of this hormone with intranuclear progesterone receptors (PR) 4.

There are two progesterone receptor isoforms: progesterone receptor A (PR-A) and progesterone receptor B (PR-B), which modify gene expression involved in cell proliferation, angiogenesis and production of epidermal growth factor (EGF) 7.

The effect of some anti-estrogenic drugs on glial tumor cells *in vitro* has aroused interest in the study of possible mechanisms of action for selective estrogen receptor modulators (SERMs), particularly tamoxifen, in these neoplasms 8,9,10,11,12.

Therefore, based upon the scarcity of studies that have investigated these receptors in glial cells, the current article aimed to conduct a literature review of the PubMed database to identify studies reporting the presence of estrogen and progesterone receptors in glial tissue over the last 12 years.

## MATERIALS AND METHODS

A PubMed database search was performed, focusing on published articles that contained quantitative studies on ER and PR expression in astrocytomas. The search was limited to the English language. Only articles published in the last 12 years were included in this review because in this period most reports involving immunohistochemistry and molecular biology on the subject were conducted. The articles were required to contain original research data for study inclusion. The search terms consisted of the following MeSH terms: “*estrogen receptor beta” OR* “*estrogen receptor alpha” OR “estrogen receptor antagonists” OR “receptors progesterone” OR “astrocytoma” OR “glioma” OR* “*glioblastoma.”*

The inclusion criteria were as follows: a) studies published in English, b) studies in which patients with brain astrocytomas were included, c) studies that investigated a correlation between hormone receptors and astrocytomas and d) studies that investigated a correlation between SERMs and astrocytomas.

To expand the scope of the search, the reference lists of all studies were inspected by two experienced authors. Studies were excluded if they were irrelevant studies, duplicate publications, articles with only abstracts available, case reports/case series, editorials, commentaries, literature reviews, letters to the editor and articles that were related to types of glial tumors other than astrocytomas.

## RESULTS

### Selected studies

**Figure 1:**
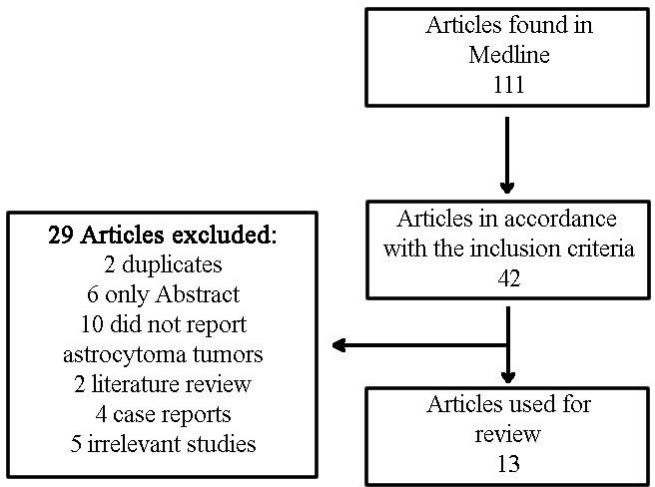
Search Flowchart.

Of the 111 titles identified in the PubMed database following the use of the keywords, only 42 satisfied the inclusion criteria. Of these 42 articles, 29 articles were excluded because 2 were duplicate articles, 6 were only available as an abstract, 10 involved tumors that were not astrocytomas, 2 were case reports, 4 were literature reviews and 5 were considered irrelevant studies by the reviewers. Thus, only 13 studies were used in the review (Figure 1).

### Study characteristics

Four articles exclusively described the presence of ERβ in glial tumors and one specifically described ERβ5 isoforms. Two studies reported the presence of ERα in astrocytomas, and only one reported the expression of both subtypes (Table 1).

Four articles reported the presence of PR in astrocytomas without specifying the subtypes. Only one study reported PR-A and another study reported PR-B (Table 1).

With increasing histological malignancy of astrocytomas, there was a decline in ERα expression described in two studies and a decrease in ERβ expression shown in another four articles. With increasing cell dedifferentiation, an increase in ERβ5 and PR expression was described in two different articles (Table 1).

Two studies showed that ER expression served as a biomarker of a good prognosis; one study reported increased ERα expression and the other reported increased ERβ expression (Table 1).

## DISCUSSION

Gliomas are tumors derived from glial cells, such as astrocytes, oligodendrocytes, microglia and ependymocytes. Gliomas are the most common type of primary central nervous system neoplasm, accounting for approximately 70-80% of all cases 2,3,4,13.

Depending to the cell of origin, these neoplasms are termed astrocytomas, oligodendrogliomas, oligoastrocytomas or ependymomas, with astrocytomas representing the most common type of glioma. According to the WHO classification, astrocytomas may be low-grade or benign and high-grade or malignant, based on the following histologic criteria: nuclear atypias, mitoses, cell proliferation and presence of necrosis 2,4,12,12,13,18.

The main risk factors for gliomas are exposure to high doses of ionizing radiation and the presence of rare genetic conditions, such as neurofibromatosis and tuberous sclerosis 15,16.

These tumors may present a variety of neurologic manifestations, such as seizures, motor and sensory deficits and changes in behavior. Complete surgical removal is usually not possible due to the infiltrative nature of the tumor and its location in critical areas of the brain 17.

According to Ho et al., who studied 21,085 glial tumors in the Netherlands, the incidence of gliomas has increased in the last 21 years, increasing from 4.9 cases per 100,000 inhabitants/year to 5.9 cases per 100,000 inhabitants/year. This rise in glioma incidence may be the result of greater awareness among physicians and the ease of performing imaging tests. As a result, larger numbers of patients are diagnosed, including those with asymptomatic tumors 16.

High-grade gliomas have an annual incidence of 3.56 cases per 100,000 inhabitants in the United States. There is a predominance in males (3:1) and gliomas mainly affect adults ranging from 40 to 60 years of age. Gliomas are among the most aggressive primary brain tumors; even when adequately treated with surgical resection, chemotherapy and radiation therapy, patients with malignant gliomas have a mean survival time of approximately 12 months. The most recent advance in treatment is the use of temozolomide as a chemotherapeutic agent. This drug has increased the mean survival time by 2.5 months and survival after two years by approximately 16% 3,4,20,21,22,23,24.

Glioblastoma multiforme (grade 4 of the WHO grading system) relapses in 100% of cases. Recurrent tumors, especially those treated previously with a combination of surgery, radiation therapy and chemotherapy, are more refractory to new therapeutic strategies 23.

Achieving prognostic improvements and more effective treatment seems to depend on the understanding and manipulation of molecular and genetic pathways that regulate the aberrant growth of these tumors. In particular, biomolecular markers have introduced further information concerning this topic in recent decades 2,21,25,26,27,28,29.

Regarding the clinical course of disease, two classes of markers have been established in oncology: prognostic and predictive markers. Prognostic markers detail the behavior of the disease regardless of the treatment adopted, while predictive markers provide information on the expected progress if a certain intervention was performed 13,26.

In a systematic review of molecular and genetic markers in the survival time of 14,678 patients with gliomas, Thuy et al. reported the existence of four main biomarkers: O-6-methylguanine methyltransferase (O-6-MGMT) methylation, isocitrate dehydrogenase 1 and 2 (IDH1/2) mutation, Ki-67/MIB1 proliferation index and loss of heterozygosity on chromosome 10/10q (LOH 10/10q) 29.

Steroid hormones exert important effects on the reproductive system, gastrointestinal tract, mammary glands, skeletal and immune systems, and even the central nervous system 5,18.

Studies have demonstrated that steroid hormones have a neuroprotective role in several neurological disorders, such as Parkinson’s disease, Alzheimer’s disease, schizophrenia and cerebrovascular accident (stroke). These neuroprotective effects include increased myelination, decreased edema, apoptosis and inflammation 18.

Hormones, mainly estrogens, may influence the development and control of brain tumor growth by interacting with their receptors or activating potentially oncogenic mediators. Estrogens seem to have a protective effect on the development of gliomas because they occur more commonly in men than in women. In women, the incidence of gliomas increases during the postmenopausal period, when estrogen levels are low 3,14,18,30.

The majority of these effects are mediated by ERα and ERβ. The former was initially characterized and cloned in 1986, and the latter was sequenced in 1996. These receptors are highly homologous, despite being products of different genes; ERα is located on chromosome 6q25.1 and ERβ is situated on chromosome 14q22-24 6.

At least five ERβ (ERβ 1-5) isoforms have been identified. These isoforms have an identical N-terminal sequence, but the amino acid sequences diverge at amino acid 469 and extend to the C-terminus. *In vitro* studies have shown different transcription activities among these isoforms 31.

The function of ERα in several neoplasms has been widely investigated, while the role of ERβ in the pathophysiology of cancer remains unknown. The presence of these receptors decreases with higher tumor grades of astrocytomas, suggesting that ERβ may play a neuroprotective role 5,6,30.

ERβ agonists and SERMs inhibit glioma tumor growth and promote tumor cell death. These findings suggest that estrogens may decrease tumor proliferation by interacting with nuclear receptors 30.

On the other hand, an *in vitro* study conducted with cell cultures by González-Arenas et al. showed that estradiol induced astrocyte growth through its interaction with ERα, recruitment of SRC-1 and SRC-2 coactivators and regulation of gene expression involved in the cell cycle, angiogenesis and metastases 32.

The loss of ERβ expression has been suggested as an important step in estrogen-dependent tumor progression. In breast tumors, high levels of ERβ receptors are associated with low-grade tumors, a favorable prognosis and a good response to tamoxifen. However, this anti-proliferative capacity has also been demonstrated in hormone-independent tumors, e.g. colon and lung neoplasms. Different mechanisms have been proposed for this anti-proliferative action, such as inhibition of ERα transcription, inhibition of phase S+G2/M and inhibition of hypoxia-inducible factor 1 (HIF1) transcription activity 6,31.

Although few studies have examined ERβ expression in brain tissue, ERβ is known to exist in neurons of the hippocampus, astrocytes, pituitary tumors and glial tumors. However, the specific function of ERβ in the pathogenesis, progression and prognosis of these neoplasms remains unknown 5,6,17.

In a study by Wenju Li et al., β5 was the most commonly found isoform among glial ERβ. In addition, ERβ expression was shown to increase with higher cell dedifferentiation, contradicting previous studies. This increased ERβ expression may have occurred as a result of hypoxia, which is commonly encountered in gliomas 31.

The actions of steroid hormones may also be mediated by coactivators, of which the family of p160 steroid receptor coactivators (SRC) has been the most widely studied. This family includes three members: SRC-1, SRC-2 and SRC-3. SRC-1 is most commonly found in brain tissue, predominantly neurons, although some astrocytes may also express this coactivator 33.

In astrocytomas, SRC-1 and SRC-3 are more abundant and typically found in the cell nucleus. In comparison, SRC-2 shows low-level expression and is most commonly located in extranuclear sites 31.

Progesterone participates in the regulation of various reproductive processes, including ovulation and sexual behavior. Nevertheless, it also influences neuronal excitability, learning and the proliferation of brain tumors, such as meningiomas, chordomas and astrocytomas 4,7.

There is abundant evidence showing that progesterone plays a neuroprotective role after injury to the central and peripheral nervous systems, limiting tissue damage or improving functional prognosis after traumatic brain injury, strokes, spinal cord injury, diabetic neuropathy and other types of acute neurologic injuries 34.

Progesterone crosses the blood-brain barrier rapidly, decreasing the inflammatory process and edema that accompanies severe traumatic brain injury 23.

The actual mechanisms responsible for these effects remain unknown. However, the major causes are the synthesis and stimulated secretion of neuroprotective substances, including neuronal growth factor (NGF), brain-derived neurotrophic growth factor (BDNF) and glial cell line-derived neurotrophic factor (GDNF) 33.

Experimental studies have shown that progesterone is capable of stimulating the infiltration and migration of astrocytes in the rat cortex. This effect may be due to various mechanisms, such as the increased expression of cell adhesion proteins, modification of the cytoskeleton and plasma membrane and even modification of voltage-dependent ion channels 35.

*In vitro* studies indicate that progesterone promotes cell proliferation in astrocytomas, as well as the expression of genes that are important for tumor growth and dissemination, e.g., cyclin D1, epidermal growth factor receptor (EGFR) and vascular endothelial growth factor (VEGF) 35,36.

However, there are several studies in the literature confirming that progesterone has anti-proliferative and apoptotic effects on ovarian, breast, endometrial and colon tumors as well as gliomas 22.

According to Atif et al., high doses of progesterone inhibit the growth of glioblastoma multiforme, both *in vitro* and in animal experiments. This effect was shown to mainly involve the inhibition of cellular proliferation and angiogenesis and the induction of apoptosis 22.

Progesterone is derived from cholesterol and exerts its effects through two major mechanisms, termed the classical and non-classical pathways. The former involves an interaction with intracellular PRs, while the latter requires the participation of membrane receptors and ion channels. These receptors are ligands of transcription factors for several genes that are involved in the metabolism, development, reproduction and progression of the cell cycle 7,37.

Two PR isoforms have been described in humans, PR-A and PR-B, and both isoforms have the same genetic origin. They are differentially expressed in various brain regions and may exert distinct functions in the same cell because they are regulated by different promoters. In general, PR-B is a stronger transcriptional activator than PR-A 7,15,35,36,38.

PRs have been found in several types of brain tumors, such as meningiomas, chordomas, craniopharyngiomas and gliomas 15,20,39.

According to some studies, PR expression increases with the histological malignancy of astrocytomas, different from that observed with ERs. In addition, there is a predominance of isoform B in high-grade gliomas 7,15,26,35,38,39,40,41.

PRs are regulated differently by estradiol and progesterone in different cells and tissues. Normally, PR function is increased (up-regulated) by estradiol and decreased (down-regulated) by progesterone 7,30.

The action of estradiol is mediated by estrogen response elements that are located in PR promoters. Progesterone causes proteolysis of PRs by means of phosphorylation 24 and this finding led us to conduct a review on ER and PR expression in astrocytomas.

Both ERα and ERβ are expressed in astrocytomas, with a predominance of isoform alpha. In the majority of studies, the presence of both ERs was shown to decrease with increasing histological tumor malignancy, suggesting a neuroprotective role, particularly of the ER beta isoform.

Both PR-A and PR-B have been reported in astrocytomas, with a predominance of the beta isoform. The presence of both PRs was shown to increase with higher tumor grades.

## AUTHOR CONTRIBUTIONS

Tavares CB reviewed the literature and wrote the manuscript. da Silva BB coordinated the study and conducted a systematic review of the manuscript. Gomes FC reviewed the literature. Costa-Silva DR reviewed the literature. Escórcio-Dourado CS reviewed the literature. Borges US reviewed the literature. Conde Junior AM reviewed the literature. Barros-Oliveira MC reviewed the literature. Sousa EB reviewed the literature. Barros LR reviewed the literature. Martins LM reviewed the literature. Facina G reviewed the literature.

## Figures and Tables

**Table 1:** Summary of the characteristics of selected studies.

Author	Year	Journal	Country	Conclusions
Batistatou, A et al.	2004	J Cancer Clin Oncol	Greece	ERβ is mainly expressed in normal astrocytes and in astrocytes of low-grade gliomas. Its presence decreases with increased malignancy of these tumors.
Batistatou A et al.	2006	Journal of Neuro-Oncology	Greece	ERβ expression is found in gliomas and oligodendrogliomas and ERβ expression tends to decrease with increased histological malignancy of the tumor. Regression models and Kaplan-Meier curves showed better prognosis and longer survival times for patients with ERβ-positive tumors.
Cabrera-Munoz E	2009	Journal of Steroid Biochemistry & Molecular Biology	Mexico	The regulation of PR expression depends on the histological grade of the astrocytoma. PR-A inhibits the effects of progesterone on growing astrocytoma cells.
Cabrera-Muñoz E et al.	2011	Current Topics in Medicinal Chemistry	Mexico	PR expression is correlated with the histological malignancy of gliomas, and PR-B is the predominant isoform in high-grade gliomas.
Sareddy G.R et al.	2012	Molecular Cancer Therapeutics	USA	ERβ expression is found in normal brain tissue and in low-grade gliomas. ERβ expression decreases with the progression of glial tumors. In high-grade gliomas, these receptors are found mainly in the cytoplasm of tumor cells. ERβ agonists inhibit the growth of gliomas in cells *in vivo*.
Hernández-Hernández O.T et al.	2012	Journal of Steroid Biochemistry & Molecular Biology	Mexico	Progesterone regulates VEGF and EGFR expression differently in astrocytoma cells by interactions with PR and SRC-1.
González-Arenas A et al.	2012	Biochimica et Biophysica Acta	Mexico	Estrogen induces the growth of human astrocytomas by interaction with ERα and recruitment of SRC-1 and SRC-3, regulating the expression of genes responsible for cell proliferation and angiogenesis.
Wenjun Lia et al.	2013	Brain Research	USA	ERβ5 is the main ERβ isoform found in gliomas. Its expression is higher in neoplasms than in normal brain tissue and increases with higher grades of cell dedifferentiation.
Jimenez J.M.D et al.	2014	Journal of Neuro-Oncology	Mexico	There is a negative correlation between ERα expression and the malignancy grade of gliomas.There is a positive correlation between ERα expression and the survival time of patients suffering from gliomas.
Liu C et al.	2014	Cancer Epidemiology	China	ERs are present in normal brain tissues and in gliomas. There is a significant reduction in ERα and ERβ expression with increased histological malignancy of the tumor.
Germán-Castelán L et al.	2014	Biomed Research International	2014	Progesterone induces the proliferation and infiltration of human anaplastic astrocytomas cells implanted in the rat motor cortex by interaction with PR.
Atif F et al.	2015	Journal of Steroid Biochemistry & Molecular Biology	USA	High doses of progesterone inhibit the *in vitro* growth of human glioblastoma multiforme, mainly by inhibiting cell growth and tumor angiogenesis and inducing apoptosis regardless of the interaction with PR.
González-Arenas A et al.	2015	General Endocrinology	Mexico	Protein C kinase α (PKCα) phosphorylates PR, and these receptors increase the genetic transcription and multiplication of astrocytomas.

ERα: Estrogen receptor alpha. ERβ: estrogen receptor beta. ERβ5: estrogen receptor beta 5. VEGF: Vascular endothelial growth factor. EGFR: epidermal growth factor receptor. GDNF: glial cell line-derived neurotrophic factor. PR: progesterone receptor. PR-A: progesterone receptor A. SRC-1: steroid receptor coactivator 1. SRC-3: steroid receptor coactivator 3.
